# Current Research on Molecular Biomarkers for Colorectal Cancer in Stool Samples

**DOI:** 10.3390/biology13010015

**Published:** 2023-12-27

**Authors:** Patricio Órdenes, Claudio Carril Pardo, Roberto Elizondo-Vega, Karina Oyarce

**Affiliations:** 1 Laboratorio de Neuroinmunología, Facultad de Medicina y Ciencia, Universidad San Sebastián, Sede Concepción, Concepción 4030000, Chile; patricio.ordenesc@docente.uss.cl (P.Ó.); claudio.carril@uss.cl (C.C.P.); 2 Laboratorio de Biología Celular, Facultad de Ciencias Biológicas, Universidad de Concepción, Concepción 4070386, Chile; relizondo@udec.cl

**Keywords:** colorectal cancer, diagnosis, methylome, epigenetics, metagenomics, transcriptomics, proteomics, metabolomics

## Abstract

**Simple Summary:**

Colorectal cancer (CRC) is a serious health problem, becoming the third most prevalent cancer and the second leading cause of cancer-related deaths. Early diagnosis is crucial as the patients have a high survival rate if the disease is detected in time. However, current CRC screening methods are either invasive or lack sensitivity and specificity, demonstrating the need to identify new ways to detect this disease. In this context, several studies have looked at small molecules present in biological fluids that can inform about the presence of tumors. Of all the biological fluids that can be analyzed, feces probably represent the fluid with the best access, as it is obtained non-invasively and has direct contact with the intestinal mucosa. This review summarizes and discusses recent advancements in the identification of potential new markers for CRC, with a focus on fecal samples.

**Abstract:**

Colorectal cancer (CRC) is one of the most diagnosed cancers worldwide, with a high incidence and mortality rate when diagnosed late. Currently, the methods used in healthcare to diagnose CRC are the fecal occult blood test, flexible sigmoidoscopy, and colonoscopy. However, the lack of sensitivity and specificity and low population adherence are driving the need to implement other technologies that can identify biomarkers that not only help with early CRC detection but allow for the selection of more personalized treatment options. In this regard, the implementation of omics technologies, which can screen large pools of biological molecules, coupled with molecular validation, stands out as a promising tool for the discovery of new biomarkers from biopsied tissues or body fluids. This review delves into the current state of the art in the identification of novel CRC biomarkers that can distinguish cancerous tissue, specifically from fecal samples, as this could be the least invasive approach.

## 1. Introduction: Colorectal Cancer Epidemiology and Risk Factors

Colorectal cancer (CRC), which includes colon and rectal cancer, is a disease that affects both men and women. It is the third most prevalent cancer and the second leading cause of all cancer-related deaths [1]. According to the Global Cancer Observatory, in 2020, there were 1.9 million new CRC cases, corresponding to 9.4% of all cancer cases, and 0.9 million deaths [1]. Moreover, the global number of new CRC cases is estimated to reach 3.2 million by 2040, based on projections of aging, population growth, and human development [2]. This increase in CRC incidence has primarily been attributed to higher incidences of exposure to modifiable risk factors, including excessive alcohol consumption, smoking, a lack of physical activity, and a western diet [2,3].

The CRC progression model posits that CRC development is slow, needing at least 10 years to manifest fully [4]. It is dependent on a series of mutations in several protooncogenes and tumor suppressor genes, such as adenomatous polyposis coli (*APC*), tumor protein *P53*, and Kirsten rat sarcoma virus (*KRAS*) [5,6,7]. It begins with genetic alterations in the intestinal mucosa that lead to the development of polyps. These polyps then grow to form precancerous masses that invade the submucosa, referred to as adenocarcinomas. When the tumor cells acquire metastatic properties that allow their dissemination, they become carcinomas [8].

CRC can be broadly classified into sporadic CRC (which represents around 65% of cases), familiar CRC (which represents 30% of cases), and hereditary CRC (representing about 5% of total cases) [5,7,9,10]. Sporadic CRC is linked to the development of cancer in people who do not carry mutations that may confer susceptibility to developing tumors. Therefore, modifiable risk factors are relevant in this type of cancer [7]. Familial CRC stands out due to the lack of an identifiable germline mutation or pattern of inheritance and a higher than expected incidence within a family [9]. Hereditary CRC, on the other hand, develops due to inherited specific mutations. The most common hereditary CRC conditions are familial adenomatous polyposis (FAP) and Lynch syndrome, known as hereditary nonpolyposis colorectal cancer (HNPCC) [11]. FAP is an autosomal dominant disorder involving mutations in the *APC* gene [12] that is responsible for the development of approximately 1% of all CRC cases and is characterized by the presence of adenomatous polyps in the colon, which can be seen from the first decade of life but become symptomatic in the second and third decades (by visible bleeding in stool). The progression of these polyps leads to the development of CRC in almost 100% of cases before age 50 [13]. On the other hand, Lynch syndrome is an autosomal dominant disorder in which several genes are mutated, namely MutL homolog 1 (*MLH1*) (50%), MutS homolog 2 (*MSH2*) (40%), MutS homolog 6 (*MSH6*) (7–10%), and PMS1 homolog 2 (*PMS2*) (5%) [14], conferring a higher risk of developing multiple types of cancer [15]. These four genes are tumor suppressor genes that normally repair errors that occur during DNA replication [16]. Lynch syndrome is responsible for at least 2–7% of CRC cases, and people who have this syndrome have a 50% chance of passing it on to their offspring [15].

Unfortunately, CRC has a poor prognosis when detected late. Hence, prevention strategies in combination with the effective early diagnosis of the disease are crucial in addressing the rising incidence over the years [17,18]. Presently, colonoscopy is considered the gold standard for the detection and prevention of CRC, as it allows for the removal of premalignant lesions in the colonic and rectal mucosa that could lead to the development of malignant tumors. However, it is an uncomfortable medical exam with low adherence (around 38%) [19,20]. Additionally, it is recommended for high-risk-profile patients, such as those with a family history of cancer or intestinal inflammatory disease [21]. Depending on the disease’s progression, many people will remain asymptomatic until visible signs, such as abdominal pain and the presence of blood in the stool, emerge. In relation to the latter, the fecal occult blood test (FOBT) has been another method used to screen for CRC [22]. However, its sensitivity and specificity are limited; therefore, it must be accompanied by other detection methods, such as colonoscopy. Thus, the development of novel, sensitive, and non-invasive diagnostic methods that allow rapid preventive screening with significant adherence are highly needed to control CRC numbers.

DNA mutations in CRC lead to transcriptional changes that affect not only the expression of these mutated genes and their targets. This, in turn, triggers alterations in signaling pathways and metabolite synthesis [23]. All these changes occurring at different molecular levels can be studied at a high resolution using omics technologies, which are defined as methodologies aiming for the universal detection of genes (genomics), DNA methylation patterns (methylome), RNAs (transcriptomics), proteins (proteomics), and metabolites (metabolomics) in complex biological samples, such as blood, serum, urine, intestinal wash, and stool [24,25,26]. The vast amount of data obtained from these methodologies can provide biomarkers able to discriminate between different CRC stages and offer specific information about the tumor that is unique to individual parties, providing the possibility of developing personalized treatments in the future. Furthermore, the application of these omics technologies in samples that are easy to obtain, such as stool, at the appropriate time, could serve as a diagnostic method to prevent people from continuing to develop CRC.

This review summarizes how various omics approaches have been used to discover new CRC biomarkers, specifically from human fecal samples, and discusses the opportunities and challenges related to the application of these technologies. This review focuses on biomarkers derived from stool samples, because they represent a non-invasive body fluid source that is easily obtained and in direct contact with the cells that form part of the precancerous and cancerous lesions in a CRC tumorigenic environment.

## 2. Genetic and Epigenetic Biomarkers from Stool for CRC Diagnosis

DNA detection in stool, due to dragged intestinal cells, can be analyzed to identify mutations and epigenetic modifications (like methylation patterns), becoming a promising method for early diagnosis. In this context, in a recent study with Chinese patients, in which fecal DNA was extracted using the immunomagnetic bead method for subsequent next-generation sequencing (NGS), higher frequencies of mutations of the *TP53*, *APC*, and *KRAS* genes were observed in CRC stool samples, very similar to tumor biopsies from the same patients [27]. Importantly, mutations in these genes disappeared from stool samples once the tumor tissue was surgically removed, demonstrating tumor specificity.

In addition, as epigenome alterations are a hallmark of cancer cells, differences in DNA methylation patterns that change the expression of oncogenes or tumor suppressor genes can be analyzed. The first studies analyzing the patterns of methylated DNA in stool from control and CRC patients were conducted by Muller, using quantitative methylation-specific PCR (qMSP), also known as MethyLight analysis (a fluorescence-based real-time PCR assay after bisulfite conversion). They identified hypermethylation in the following genes: Secreted Frizzled Related Protein 2 and 5 (*SFRP2* and *SFRP5*), progesterone receptor (*PGR*), calcitonin-related polypeptide alpha (*CALCA*), and insulin-like growth factor binding protein 2 (*IGFBP2*) [28]. *SFRP2* hypermethylation was subsequently confirmed by other studies in stool samples from patients with CRC, adenomas, and advanced precancerous stages. Therefore, this marker would be a candidate in CRC screening tests [29,30,31,32,33,34,35,36].

Other genes under consideration as a CRC biomarker for American and Chinese patients are *Wnt* inhibitory factor 1 (*WIF-1*) [36,37] and vimentin (*VIM*), which codifies an intermediate filament protein that is not methylated in normal colonic epithelial cells but becomes methylated in 53–83% of CRC tissues [38]. *VIM* gene methylation was particularly elevated in adenoma and CRC stool samples, as analyzed by qMSP [37,38,39,40,41,42,43,44].

An additional gene found to exhibit hypermethylation in stool samples from individuals with high-grade dysplasia, adenomas, and CRC is *N-Myc* downstream-regulated gene 4 (*NDRG4*), which codifies a protein involved in cell cycle regulation and differentiation [33,39,45,46,47]. Other genes that show increased methylation in their promoter regions are tissue factor pathway inhibitor 2 (*TFPI2*) [33,36,39] and bone morphogenetic protein 3 (*BMP3*) [33,39,45,48]. The downregulation of *BMP3* could be involved in the early stages of CRC tumorigenesis [49].

Based on mutations found in some CRC-related genes and methylation alterations, a panel of CRC biomarkers from stool DNA, known as multi-target stool DNA (mt-sDNA), was created in 2014 for the screening of CRC in patients with a high risk of developing the disease [45,50]. This test, known as COLOGUARD, evaluates seven *KRAS* mutation markers, *NDGR4* and *BMP3* methylation, and hemoglobin as a control in stool samples. Hemoglobin, a protein found in red blood cells, was incorporated into the kit because its presence indicates intestinal bleeding, which is one of the clinical signs of adenoma and CRC. This mt-sDNA has been widely used as a screening method in countries such as the USA, to detect advanced CRC neoplasms [51]. Although this test has high sensitivity (90%) for the detection of multiple cancerous lesions and advanced stages of CRC, it only detects about 42% of polyps, compared to 92% by colonoscopy. Another limitation is the high rate of false positives, which is around 13% and increases with age [51]. Therefore, a colonoscopy must fallow this examination, especially if polyps are suspected.

Other studies using stool samples from Korean and Chinese patients have detected the hypermethylation of syndecan 2 (*SDC2*) gene promoter [34,46,52,53,54,55,56]. Meanwhile, a report from Taiwan found increased methylation in genes of alcohol dehydrogenase iron containing 1 (*ADHFE1*), *SDC2*, and protein phosphatase 2 regulatory subunit B′gamma (*PPP2R5C*) in CRC samples [57]. Another study using TaqMan qMSP in fecal samples from Chinese patients with CRC has determined differences in methylation for other genes, such as collagen type IV alpha 1 and alpha 2 chain (*COL4A1*, *COL4A2*), T-cell leukemia homeobox protein 2 (*TLX2*), and integrin subunit alpha 4 (*ITGA4*) [58]. The hypermethylation of *ITGA4* was also previously reported in the Korean population [30].

On the other hand, a study with stool DNA from Italian patients using MethyLight and digital PCR technology observed hypermethylation in the CpG islands of the genes glutamate ionotropic receptor AMPA type subunit 4 (*GRIA4*) and vasoactive intestinal peptide receptor 2 (*VIPR2*), with similar results in biopsy samples from the same patients, which were also validated at the mRNA and protein level [59].

As can be seen, some methylation biomarkers exhibit good consistency in different ethnicities. All the genetic biomarkers described above are listed in Table 1. The order in which each biomarker appears in the text is followed without any particular hierarchy.

## 3. Microbiome Analysis of Stool for CRC Diagnosis through Metagenomics

Microbiome is a term that describes the genomes of all microorganisms, symbiotic and pathogenic, living in and on all vertebrates [60]. Growing evidence indicates that the microbiome is associated with the development of CRC as the abundance of specific types of bacteria has been demonstrated and they are thought to actively participate in CRC development by secreting pro-inflammatory factors and metabolites [61,62]. Thus, the analysis of the microbiome from stool by metagenomics, a field that investigates the genetic material found in a determined microenvironment, can be used to determine the enrichment or loss of specific bacteria in CRC patients. These differentially present bacteria can serve as early CRC biomarkers and novel targets.

Metagenomics can be performed untargeted, which means that the entirety of the DNA will be sequenced, or targeted to specific genes that are highly conserved through evolution, so their nucleotide variation allows for the identification of specific genera. The most common one is ribosomal RNA subunit 16S (*16S rRNA*).

In this regard, a metagenomic study on Chinese and Danish patients, with later validation by qRT-PCR and comparison with public metagenomic data from previous French and Austrian cohorts, identified the enrichment of the genes butyryl-CoA dehydrogenase from *Fusobacterium nucleatum*; and RNA polymerase subunit β (*rpoB*) from *Parvimonas micra* in samples from CRC patients. Importantly, these findings were shared by all groups despite belonging to different ethnicities [63].

*P. micra* has also been detected as enriched in fecal samples from CRC patients from Swedish patients using qPCR [64] and Malay, Chinese, and Indian patients, along with *Peptostreptococcus stomatis*, *F. nucleatum*, and *Akkermansia muciniphila* [65]. Meanwhile, *F. nucleatum* has been largely identified as an enriched bacterium in samples from German [66,67], Japanese [68], Swedish [64], and Chinese patients with CRC [69].

Another study that analyzed metagenomic data, this time comprising 16S rRNA gene sequencing data from 19 independent studies with Chinese, American, Irish, Italian, Canadian, and Spanish patients, revealed the presence of harmful genera in the samples of patients with CRC, such as *F. nucleatum* and *Echerichia/Shigella*, among others [70]. On the other hand, a more recent study with fecal samples from Chinese CRC patients detected an increase in bacteria such as *Coriobacteriaceae bacterium*, *P. micra*, *F. nucleatum*, *Gemella morbillorum*, *Citrobacter portucalensis*, *Alloprevotella* sp., and *Shigella sonei* [71].

Other studies have directed their attention to differentiating bacteria strains between adenoma and CRC, with the aim of facilitating early disease detection. For instance, a study with Iranian patients determined that *F. nucleatum, Enterococcus fecalis, Streptococcus bovis, Enterotoxigenic Bacteroides fragilis,* and *Porphyromonas* spp. were enriched in samples from adenoma compared to the controls [72]. Wu and colleagues, on the other hand, used public sequencing data for gene 16S rRNA from the stool of American and Canadian patients to define a set of bacteria that was later searched in Chinese stool samples. They observed that *P. micra*, *Clostridium scindens*, *Blautia* sp., *Eubacterium coprostanoligenes* group sp., *Ruminococaceae UCG-002* sp., and *Porphyromonas* sp., among others, were enriched in CRC compared to adenoma, while *Bacteroides dorei, Eubacterium ruminantium, Erysipelatoclostridium ramosum,* and *Lachnospira pectinoschiza* were particularly enriched in adenoma but not CRC, so they could be included in an early CRC diagnosis panel [73].

Recent metagenomic analyses have expanded their scope by incorporating patient age and the incidence of virulence factors, hypothesizing that the appearance of these factors increases the probability of early cancer development. Results reveal the enrichment of colibactin and FadA genes, as well as an increase in the presence of *F. nucleatum* bacteria in CRC patients, although with limited statistical power given the few samples of young patients [74].

Overall, *F. nucleatum* and *P. micra* are the bacteria most consistently enriched in CRC across different studies, with only one study also detecting *F. nucleatum* in the adenoma stage. Although more studies are needed to validate its presence at the early stages of CRC development, *F. nucleatum* is being considered as a promoter of carcinogenesis as it has been shown to increase the expression of proteins that promote cell cycle progression through the activation of proinflammatory pathways [75], and it also promotes macrophage infiltration into tumors and increases angiogenesis and immune system evasion [76].

The metagenomics biomarkers are summarized in Table 2, in the same order in which each biomarker appears in the text, without any other particular hierarchy.

Although interesting results have been obtained after statistical modeling, and good candidates have emerged as CRC biomarkers, gut dysbiosis is known to be a feature of a wide range of diseases, such as diabetes, obesity, and even neurodegenerative disorders. Thus, enriched bacteria found in CRC, in the context of control patients with no other diseases, are not likely to be specific for CRC. Studies including patients for other pathological conditions are needed to ensure that these candidates can be used in population-based screening for CRC.

## 4. Analysis of RNA Molecules in Stool from CRC Patients

The analysis of differentially expressed genes in both control and CRC patients has emerged as an approach to identifying clinically relevant biomarkers. One of the pioneer studies in establishing protocols for the identification of transcript-based biomarkers for CRC in stool samples was conducted by Ahmed et al., 2004. The authors of this work found, through qRT-PCR, the overexpression of guanylyl cyclase (*GCC*) carcinoembryonic antigen (*CEA*), PYRIN-containing Apaf-1-like proteins (*PYPAF5*), histone family member 1 (*H1F1*), human T-cell leukemia virus type I binding protein 2 (*TAX1BP2*), olfactory receptor family 2 subfamily 1 putative (*OR2I4P*) and subfamily A member 7 (*OR2A7*), and found in inflammatory zone 1 (*FIZZ1*) in stool samples from American patients with CRC [77].

In a study conducted with Japanese stool samples, it was determined that the expression of RNAs *CEA*, beta-2 microglobulin (*B2M*), E-cadherin, *CD45*, and cyclooxygenase-2 (*COX-2*) was elevated in CRC patients. Interestingly, the level of *COX-2* expression was found to correlate well with the tumor size [78]. In another study on the Japanese population, an increase in integrin alpha 6 (*ITGA6*) was found in CRC patients, in comparison with controls [79]. More recent studies with Japanese and Canadian CRC patients have also revealed the increased expression of the following transcripts: growth arrest and DNA damage inducible beta (*GADD45B*), integrin subunit alpha 2 (*ITGA2*), MYB proto-oncogene like 2 (*MYBL2*), *MYC*, prostaglandin-endoperoxide synthase 2 (*PTGS2*), and *S100A4* [20], while *CEA* cell adhesion molecule 5 (*CEACAM5*), integrin alpha 6 (*ITGA6*), and MET transcriptional regulator (*MACC1*) showed increased expression in patients with adenoma [20,80]. Thus far, the RNA biomarker *CEA* and *ITGA6* are detected more consistently in samples from populations with different ethnicities, suggesting that they could be good candidates for CRC screening. More studies are needed to confirm that *ITGA6* is enriched more specifically in the adenoma stage.

Other molecules that have been the subject of study are microRNAs (miRNAs), small non-coding RNA molecules that participate in the post-transcriptional regulation of gene expression [81]. Studies carried out by Ahmed et al. detected seven miRNAs significantly upregulated in stool samples from American patients with CRC, compared to controls, namely miR-92, miR-106a, miR-96, miR-203, miR-326 miR-20a, and miR-21, and seven miRNAs significantly downregulated: miR-320, miR-126, miR-484-5p, miR-143, miR-145, miR-16, and miR-125b [82]. Moreover, twelve miRNAs increased in CRC, compared to adenoma, which could be used to discriminate between these two stages: miR-7, miR-17, miR-20a, miR-21, miR-92a, miR-96, miR-106a, miR-134, miR-183, miR-196a, miR-199a-3p, and miR-214. Meanwhile, eight miRNAs were decreased in CRC: miR-9, miR-29b, miR-127-5p, miR-138, miR-143, miR-146a, miR-222, and miR-938 [83]. Among these miRNAs, miRNA-21 has been found to be over-regulated in patients with CRC by other studies for the Iranian [84] and Korean populations [85]; in addition, miR-92a has been found enriched in stool samples from CRC patients [85,86].

Other studies have shown, in Chinese patients, that miRNA-223 is overexpressed in CRC [86,87], while, in contrast, Zhu et al. detected lower expression in CRC samples [88].

The only study so far with the Hispanic population found that miR-421, miR-130b-3p, and miR-27a-3p were increased in stool samples from patients with adenomas and CRC [89], but none of these have been previously reported by other studies.

Finally, there is only a single study that has analyzed differences in long non-coding RNAs (lncRNA) through qRT-PCR in stool samples from Iranian CRC patients, observing an increased level of prostate cancer-associated transcript 1 (*PCAT1*), colon cancer-associated transcript 1 and 2 (*CCAT1*, *CCAT2*), tumor suppressor candidate 7 (*TUSC7*), *H19*, HOX antisense intergenic RNA (*HOTAIR*), highly upregulated in liver cancer (*HULC*), phosphatase and tensin homolog PSEUDOGEN 1 (*PTENP1*), metastasis-associated lung adenocarcinoma transcript 1 (*MALAT1*), and maternally expressed 3 (*MEG3*) [90]. More lncRNA studies are needed to fully determine the diagnosis potential of these molecules.

Although a considerable number of studies have found significant differences in the expression of RNAs and miRNAs between control and adenoma/CRC patients, there is no standardized RNA panel yet that reliably serves for CRC diagnosis. However, the most promising miRNAs so far are miRNa-21 and miRNA-92a. It is important to consider that differences in RNA molecules found so far might be due to differences between populations with particular ethnic characteristics or lifestyles, such as diet. Furthermore, each research group uses different protocols for sample processing and RNA extraction, reducing the possibility of a standardized protocol. Therefore, further investigations of the methodological–clinical applicability concerning miRNAs as markers of adenoma and CRC should be considered.

All the summarized transcripts are listed in Table 3, in the same order in which each biomarker appears in the text, without any other particular hierarchy. 

## 5. Proteomic Analysis of Stool in CRC

Proteomics is the large-scale study of the structure and function of the complete set of proteins from a biological context, including how they function and interact with each other [91]. In the context of cancer, proteomics studies focus on the differential expression of proteins between cells in a tumorigenic environment compared to normal profiles. Some of the most used techniques for the identification of protein biomarkers in CRC are two-dimensional gel electrophoresis combined with liquid chromatography/mass spectrometry (LC-MS) and some derivations. One study on stool samples from German patients found eleven proteins that had increased expression in CRC compared to control samples: azurocidin 1 (AZU1), complement component 3 (C3) and 5 (C5), cytidine deaminase (CDA), myeloperoxidase (MPO), fibronectin 1 (FN1), lactotransferrin (LTF), haptoglobin (HP), hemoglobin subunit beta (HBB) and subunit alpha1 (HBA1), and retinol binding protein 4 (RBP4). Meanwhile, five proteins discriminated advanced adenoma from control samples: AZU1, hemopexin (HPX), LTF, MPO, and serpin family F member 2 (SERPINF2) [92]. It is worth highlighting that the vast majority of these differentially expressed proteins are related to hemoglobin homeostasis, indicating the presence of blood. This correlates well with the use of current FOBT, which searches for the presence of hemoglobin; however, because intestinal bleeding can also be caused by inflammatory bowel disease and hemorrhoids, they are not necessarily specific for CRC. On the other hand, complement component proteins are indicative of inflammation and complement activation has been shown to promote colitis-associated CRC [93], so the presence of C3 and C5 could indicate a higher risk of developing CRC.

Subsequently, another study with stool samples from German and Dutch patients identified increased levels of HP, lysosomal-associated membrane protein 1 (LAMP1), spectrin repeat containing nuclear envelope protein 2 (SYNE2), and annexin A6 (ANXA6) in adenoma stool samples compared to controls, while HP, leucine-rich α-2 glycoprotein 1 (LRG1), RBP4, and FN1 were increased in CRC samples [94]. In congruence with the previous report, these results support HP, RBP4, and FN1 as candidates for CRC screening tests; however, because both studies were conducted in similar populations, it remains to be determined whether these findings will be observed in other ethnicities.

It is noteworthy that the differentially expressed proteins found in stool samples could be derived from the cytoplasm of ruptured cells, the pool of secreted proteins, or extracellular vesicles (EVs) released by the tumoral environment. In this context, we found only one study that has looked for potential CRC biomarkers in fecal EVs (fEVs). Zhang et al., 2023 showed, by Western blot, immunogold transmission electron microscopy, immunofluorescence, and ELISA, that the levels of the proteins CD147 and cell surface A33 antigen on fEVs were higher in CRC patients, compared to healthy donors, with no significant variation across the different stages of CRC [95].

All the proteins summarized are listed in Table 4, in the same order in which each biomarker appears in the text, without any other particular hierarchy.

## 6. Metabolomic Analysis of Stool in CRC

Metabolomics can be defined as the comprehensive analysis of the intermediate metabolites as well as end products of a metabolic pathway [96]. Tumor cell metabolism is known to change to adapt to the demands of dysregulated proliferation and metastasis; therefore, metabolomics could be useful to identify these metabolic changes between healthy and CRC patients with high sensitivity [25]. Metabolomics of stool samples in CRC patients, by either nuclear magnetic resonance (NMR) spectroscopy or high-resolution liquid chromatography (HPLC), shows alterations in many types of metabolites, from glucose and glutamate to fatty acids, amino acids, and nucleotides [25,69,97]. One of the first studies to determine the metabolomic profile of stool from CRC American patients detected increased levels of amino acids and their metabolic derivatives, as well as monosaccharides fructose, mannose, and galactose, vitamins, and their cofactors [98]. Interestingly, the authors compared the metabolites found in stool with colonic mucosa from CRC patients, finding seven metabolites in common: an increase in metabolites derived from amino acids alpha-hydroxyisovalerate, isovalerate, and valerate; a metabolic product from NAD degradation, N1-methyl-2-pyridine-5-carboxamide; and a decrease in secondary bile acid metabolites 7-ketodeoxycholate, deoxycholate, and dipeptide tryptophylglycine [98].

A study in Chinese patients revealed an increase in polyamine cadaverine, several amino acids, urea, and butanedioic acid in CRC patients [69]. When the metabolites were correlated with metagenomic data from the same samples, the most abundant metabolites in CRC were cadaverine and putrescine, reaching sensitivity equivalent to that of FOBT [69]. Another study in Chinese patients detected increased levels of some amino acids, lactate, glutamate, and succinate, and lower levels of butyrate, propionate, and acetate, in the fecal samples from CRC [99].

On the other hand, a study with stool samples from Italian patients showed decreased levels of galactose, acetate, xylose, isobutyrate, and 3-hydroxyphenylacetate, and higher levels of glycerol and phenylalanine, in CRC. Meanwhile, lower amounts of 3-hydroxyphelylacetate, butyrate, acetate, propionate, and isobutyrate were detected in adenoma samples [100].

Finally, two studies in Spanish patients have detected changes in metabolites from lipidic pathways in CRC samples, such as increased levels of cholesterol esters ChoE(18:1), ChoE(18:2), ChoE(20:4) [19,101], sphingomyelins SM(d18:1/23:0), SM(42:3) [101], and phosphatidylethanolamine PE(16:0/18:1) [19].

As is noticeable, most of these metabolites are associated with increased demands on anabolic pathways, particularly amino acids, which can be easily related to a higher proliferation rate but also could be explained by other metabolic alterations or dietary patterns. Therefore, CRC-specific metabolites have not been described yet, which is understandable due to how recently these types of studies have been developed.

The most relevant metabolites have been summarized in Table 5, in the same order in which each biomarker appears in the text, without any other particular hierarchy.

## 7. Conclusions

The current diagnostic methods for CRC, which include colonoscopy and flexible sigmoidoscopy, represent invasive and low-adherence exams that are usually recommended when bleeding has been detected by FOBT, a screening test with low and variable sensitivity, making timely diagnosis very difficult. This has led researchers to search for molecular biomarkers for the detection of polyps, adenomas, and CRC in non-invasive fluids such as stool. In this context, the advancement of omics technologies has allowed the identification of differentially present molecules, which some studies have further validated, representing an interesting opportunity to improve or complement early CRC diagnosis, establishing more personalized treatments and increasing life expectancy. Despite this promising progress, to validate the large number of biomarkers found, the gold standard that is colonoscopy must continue to be used, and as long as there is no large-scale validation, we probably cannot do without this confirmatory examination in the near future. However, the aim of identifying CRC biomarkers in feces is to have new tools for population screening that are quick, safe, and suitable for younger populations, where CRC has increased in recent years, allowing for early detection.

## 8. Future Directions

**Figure 1:**
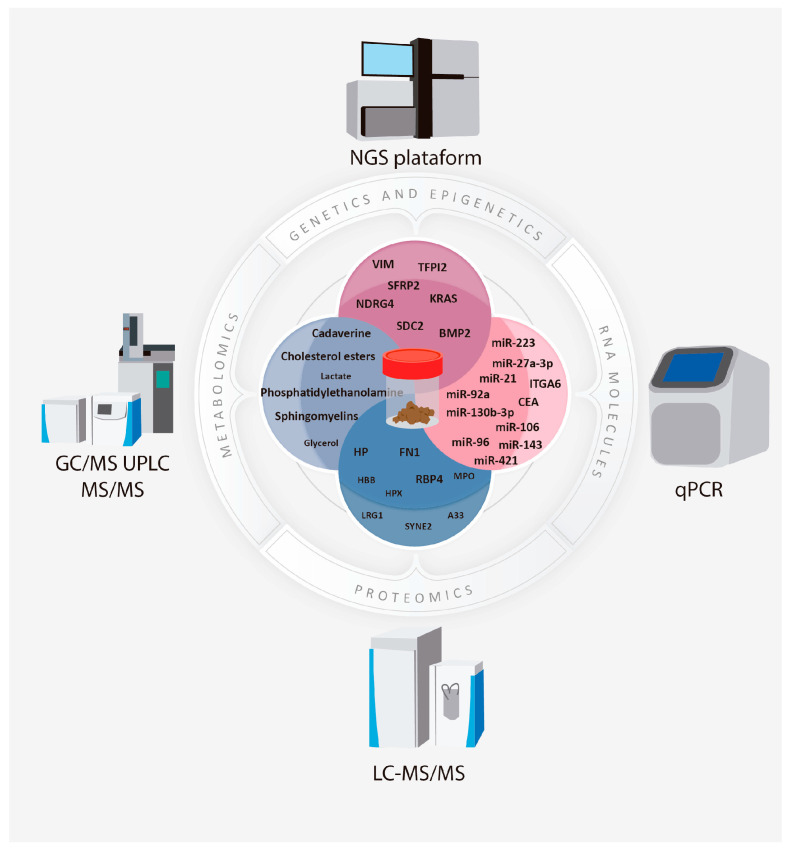
Summary of the methods used for discovery of CRC molecular biomarkers in stool, indicating the ones with higher reproducibility among studies analyzed.

Although several studies have provided promising candidates as biomarkers for adenoma and CRC, at the level of DNA, RNA, protein, and even metabolites (Figure 1), for most of them, very little consistency is observed between studies. These differences could be explained by population heterogeneity and the lack of standardization in sample processing. In addition, most of the studies have been carried out in Asian, American, and European populations, so further validation of these results is required in African and Latin American countries, where no studies applying high-resolution omics technologies for CRC are reported.

While most of the literature thus far has focused on genomic and transcriptomic approaches, very few studies have reported potential biomarkers at the protein level. We believe that it is necessary to continue the search for CRC biomarkers through proteomics approaches, given that they may be more readily validated and later implemented in clinical settings, particularly when contemplating the subsequent use of rapid tests based on ELISA.

## Figures and Tables

**Table 1:** Genetic and epigenetic biomarkers for CRC detection from stool samples.

Gene	Study Population	Methodology	References
**Mutation**			
*TP53*	Chinese	NGS	[27]
*APC*	Chinese	NGS	[27]
*KRAS*	Chinese	NGS	[27]
**Hypermethylation**			
*SFRP2*	Austrian	MethyLight	[28]
	Korean	Methylation-specific PCR	[30]
	Chinese	Methylation-specific PCR	[31]
	Austrian	MethyLight	[32]
	Chinese	MethyLight	[34]
	Chinese	MethyLight	[35]
	Chinese	Methylation-specific PCR	[36]
	Korean	Methylation-specific PCR	[33]
	Iranian	Methylation-specific PCR	[29]
*VIM*	American	Methylation-specific PCR	[38]
	American	Methylation-specific PCR	[37]
	Chinese	Methylation-specific PCR	[43]
	Chinese	Methylation-specific PCR	[44]
	American	Methylation-specific PCR	[39]
*NDRG4*	American	Methylation-specific PCR	[39]
	American; Canadian	Methylation-specific PCR	[45]
	Chinese	Methylation-specific PCR	[46]
	Belgian	Methylation-specific PCR	[47]
	Korean	Methylation-specific PCR	[33]
	American	Methylation-specific PCR	[48]
*BMP3*	American	Methylation-specific PCR	[39]
	American; Canadian	Methylation-specific PCR	[45]
	American	Methylation-specific PCR	[48]
	Korean	Methylation-specific PCR	[33]
*SDC2*	Chinese	MethyLight	[34]
	Chinese	Methylation-specific PCR	[46]
	Chinese	MethyLight	[52]
	Korean	Methylation-specific PCR	[53]
	Chinese	Methylation-specific PCR	[56]
	Chinese	Methylation-specific PCR	[55]
	Taiwanese	Methylation-specific PCR	[57]
	Chinese	Methylation-specific PCR	[54]
*COL4A1*	Chinese	Methylation-specific PCR	[58]
*COL4A2*	Chinese	Methylation-specific PCR	[58]
*TLX2*	Chinese	Methylation-specific PCR	[58]
*ITGA4*	Chinese	Methylation-specific PCR	[58]
	Korean	Methylation-specific PCR	[30]
*WIF1*	Chinese	Methylation-specific PCR	[36]
	American	Methylation-specific PCR	[37]
*GRIA4*	Italian	MethyLight	[59]
*VIPR2*	Italian	MethyLight	[59]
*SFRP5*	Austrian	MethyLight	[28]
*PGR*	Austrian	MethyLight	[28]
*CALCA*	Austrian	MethyLight	[28]
*IGFBP2*	Austrian	MethyLight	[28]
*TFPI2*	American	Methylation-specific PCR	[39]
	Korean	Methylation-specific PCR	[33]
	Chinese	Methylation-specific PCR	[36]
*p16*	Korean	Methylation-specific PCR	[30]
*KRAS*	Chinese	MethyLight	[34]
	American	Methylation-specific PCR	[39]
*ALX4*	American	Methylation-specific PCR	[37]
*OMSR*	Chinese	Methylation-specific PCR	[36]
*ADHFE1*	Taiwanese	Methylation-specific PCR	[57]
*PPP2R5C*	Taiwanese	Methylation-specific PCR	[57]
*SHOX2*	Chinese	Methylation-specific PCR	[54]

**Table 2:** Metagenomic studies from stool samples for CRC detection.

Bacteria	Study Population	CRC Stage	References
*Fusobacterium nucleatum*	Chinese, Danish, French, Austrian	CRC	[63]
	German	CRC	[66]
	German	CRC	[67]
	Japanese	CRC	[68]
	Swedish	CRC	[64]
	Chinese	CRC	[69]
	Chinese, American, Irish, Italian, Canadian, Spanish	CRC	[70]
	Chinese	CRC	[71]
	Iranian	AD	[72]
	Austrian, Canadian, Chinese, German, French, Indian, Italian, Japanese, American	CRC	[74]
*Parvimonas micra*	Chinese, Danish, French, Austrian	CRC	[63]
	Swedish	CRC	[64]
	Malay, Chinese and Indian	CRC	[65]
	Chinese	CRC	[71]
	American, Canadian, Chinese	CRC	[73]
*Peptostreptococcus stomatis*	Malay, Chinese and Indian	CRC	[65]
*Akkermansia muciniphila*	Malay, Chinese and Indian	CRC	[65]
*Echerichia/Shigella*	Chinese, American, Irish, Italian, Canadian, Spanish	CRC	[70]
*Coriobacteriaceae bacterium*	Chinese	CRC	[71]
*Gemella morbillorum*	Chinese	CRC	[71]
*Citrobacter portucalensis*	Chinese	CRC	[71]
*Alloprevotella* sp.	Chinese	CRC	[71]
*Shigella sonei*	Chinese	CRC	[71]
*Enterococcus fecalis*	Iranian	AD	[72]
*Streptococcus bovis*	Iranian	AD	[72]
*Enterotoxigenic Bacteroides fragilis*	Iranian	AD	[72]
*Porphyromonas* sp.	Iranian	AD	[72]
	American, Canadian, Chinese	CRC	[73]
*Clostridium scindens*	American, Canadian, Chinese	CRC	[73]
*Blautia* sp.	American, Canadian, Chinese	CRC	[73]
*Eubacterium coprostanoligenes group* sp.	American, Canadian, Chinese	CRC	[73]
*Ruminococaceae UCG-002* sp.	American, Canadian, Chinese	CRC	[73]
*Bacteroides dorei*	American, Canadian, Chinese	AD	[73]
*Eubacterium ruminantium*	American, Canadian, Chinese	AD	[73]
*Erysipelatoclostridium ramosum*	American, Canadian, Chinese	AD	[73]
*Lachnospira pectinoschiza*	American, Canadian, Chinese	AD	[73]

**Table 3:** RNA molecules from stool samples for CRC detection. Arrows pointing upwards indicate an increase in their expression levels, while arrows pointing downward indicate decrease expression.

Potential Biomarker	Study Population	CRC Stage	References
mRNA			
*GCC*	American	CRC	[77]
*CEA*	American	CRC	[77]
	Japanese	CRC	[78]
*PYPAF5*	American	CRC	[77]
*H1F1*	American	CRC	[77]
*TAX1BP2*	American	CRC	[77]
*OR2I4P*	American	CRC	[77]
*OR2A7*	American	CRC	[77]
*FIZZ1*	American	CRC	[77]
*B2M*	Japanese	CRC	[78]
*E-CAD*	Japanese	CRC	[78]
*CD45*	Japanese	CRC	[78]
*COX-2*	Japanese	CRC	[78]
*ITGA6*	Japanese	AD, CRC	[79]
	Japanese, Canadian	AD	[20,80]
*GADD45B*	Japanese, Canadian	CRC	[20]
*ITGA2*	Japanese, Canadian	CRC	[20]
*MYBL2*	Japanese, Canadian	CRC	[20]
*MYC*	Japanese, Canadian	CRC	[20]
*PTGS2*	Japanese, Canadian	CRC	[20]
*S100A4*	Japanese, Canadian	CRC	[20]
*CEACAM5*	Japanese, Canadian	AD	[20]
*MACC1*	Japanese, Canadian	AD	[20]
miRNA			
miR-7	American	↑CRC	[83]
miR-9	American	↓CRC	[83]
miR-16	American	↓CRC	[82]
miR-17	American	↑CRC	[83]
miR-20a	American	↑CRC	[82]
	American	↑CRC	[83]
miR-21	American	↑CRC	[82]
	American	↑CRC	[83]
	Iranian	↑CRC	[84]
	Korean	↑CRC	[85]
miR-27a-3p	Spanish	↑AD ↑CRC	[89]
miR-29a	Chinese	↓CRC	[88]
miR-29b	American	↓CRC	[83]
miR-92	American	↑CRC	[82]
miR-92a	Taiwanese	↑CRC	[86]
	Korean	↑CRC	[85]
	American	↑CRC	[83]
miR-96	American	↑CRC	[82]
	American	↑CRC	[83]
miR-106a	American	↑CRC	[82]
	American	↑CRC	[83]
miR-125b	American	↓CRC	[82]
miR-126	American	↓CRC	[82]
miR-127-5p	American	↓CRC	[83]
miR-130b-3p	Spanish	↑AD ↑CRC	[89]
miR-134	American	↑CRC	[83]
miR-138	American	↓CRC	[83]
miR-143	American	↓CRC	[82]
	American	↓CRC	[83]
miR-145	American	↓CRC	[82]
miR-183	American	↑CRC	[83]
miR-196a	American	↑CRC	[83]
miR-199a-3p	American	↑CRC	[83]
miR-203	American	↑CRC	[82]
miR-214	American	↑CRC	[83]
miR-222	American	↓CRC	[83]
miR-223	Taiwanese	↑CRC	[86]
	Chinese	↑CRC	[87]
	Chinese	↓CRC	[88]
miR-224	Chinese	↓CRC	[88]
miR-320	American	↓CRC	[82]
miR-326	American	↑CRC	[82]
miR-421	Spanish	↑AD ↑CRC	[89]
miR-451	Chinese	↑CRC	[87]
miR-484-5p	American	↓CRC	[82]
miR-938	American	↓CRC	[83]
lncRNA			
*PCAT1*	Iranian	↑CRC	[90]
*CCAT1*	Iranian	↑CRC	[90]
*CCAT2*	Iranian	↑CRC	[90]
*TUSC7*	Iranian	↑CRC	[90]
*H19*	Iranian	↑CRC	[90]
*HOTAIR*	Iranian	↑CRC	[90]
*HULC*	Iranian	↑CRC	[90]
*PTENP1*	Iranian	↑CRC	[90]
*MALAT1*	Iranian	↑CRC	[90]
*MEG3*	Iranian	↑CRC	[90]

**Table 4:** Proteomic studies from stool samples for CRC detection. Arrows pointing upwards indicate an increase in their expression levels, while arrows pointing downward indicate decrease expression.

Potential Biomarker	Study Population	CRC Stage	References
AZU1	German	↑AD ↑CRC	[92]
C3	German	↑CRC	[92]
C5	German	↑CRC	[92]
CDA	German	↑CRC	[92]
MPO	German	↑AD ↑CRC	[92]
FN1	German	↑CRC	[92]
	German, Dutch	↑CRC	[94]
LTF	German	↑AD ↑CRC	[92]
HP	German	↑CRC	[92]
	German, Dutch	↑AD ↑CRC	[94]
HBB	German	↑CRC	[92]
HBA1	German	↑CRC	[92]
RBP4	German	↑CRC	[92]
	German, Dutch	↑CRC	[94]
HPX	German	↑AD	[92]
SERPINF12	German	↑AD	[92]
LAMP1	German, Dutch	↑AD	[94]
SYNE2	German, Dutch	↑AD	[94]
ANXA6	German, Dutch	↑AD	[94]
LRG1	German, Dutch	↑CRC	[94]
CD147	Chinese	↑CRC	[95]
A33	Chinese	↑CRC	[95]

**Table 5:** Metabolomic studies from stool samples for CRC detection. Arrows pointing upwards indicate an increase in their expression levels, while arrows pointing downward indicate decrease expression.

Potential Biomarker	Study Population	CRC Stage	References
Alpha-hydroxyisovalerate	American	↑CRC	[98]
Isovalerate	American	↑CRC	[98]
Valerate	American	↑CRC	[98]
N1-methyl-2-pyridine-5-carboxamide	American	↑CRC	[98]
7-ketodeoxycholate	American	↓CRC	[98]
Deoxycholate	American	↓CRC	[98]
Tryptophylglycine	American	↓CRC	[98]
Cadaverine	Chinese	↑CRC	[69]
Putrescine	Chinese	↑CRC	[69]
Alanine	Chinese	↑CRC	[99]
Lactate	Chinese	↑CRC	[99]
Glutamate	Chinese	↑CRC	[99]
Succinate	Chinese	↑CRC	[99]
Glycerol	Italian	↑CRC	[100]
Phenylalanine	Italian	↑CRC	[100]
3-hydroxyphenyl acetate	Italian	↓CRC	[100]
Galactose	Italian	↓CRC	[100]
Acetate	Italian	↓AD ↓CRC	[100]
	Chinese	↓CRC	[99]
Xilose	Italian	↓CRC	[100]
Isobutyrate	Italian	↓AD ↓CRC	[100]
Butyrate	Italian	↓AD	[100]
	Chinese	↓CRC	[99]
Propionate	Italian	↓AD	[100]
	Chinese	↓CRC	[99]
3-hydroxyphenyl acetate	Italian	↓AD	[100]
Cholesterol esters	Spanish	↑CRC	[19]
	Spanish	↑CRC	[101]
Sphingomyelins	Spanish	↑CRC	[19]
	Spanish	↑CRC	[101]
Phosphatidylethanolamine	Spanish	↑CRC	[19]
	Spanish	↑CRC	[101]

## Data Availability

Not applicable.
